# 

**Published:** 1867-10

**Authors:** 


### CONTENTS.

### ORIGINAL COMMUNICATIONS.

PAGE.

ARTICLE I.—Report of a Case of Typhoid Fever, with Remarks. By CHARLES PINCKNEY, M. D., of Atlanta, Ga .... 329
ARTICLE II.—Abstract of Minutes of the Atlanta Medical Society .... 333
ARTICLE III.—A Case of Vulvitis in a Little Girl, Terminating Fatally; with Remarks. By A. GIVEN, M. D., of Louisville, Ky .... 336
ARTICLE IV.—Dublin Correspondence .... 342

### SELECTIONS.

On the Internal Administration of Chloroform in Pernicious Fever .... 349
Bloodletting Then and Now .... 352
"Missed Labor" .... 358

### EDITORIAL AND MISCELLANEOUS.

Caution and Simplicity, the True Test of Excellence in the Practice of Medicine .... 363
Omission .... 368
Our European Correspondence .... 369
Prof. W. S. Armstrong .... 369
Prof. D. W. Yandell .... 369
What will ensure a high standard of Medical Knowledge? .... 370
Grubs in the Face—Acne Punctata .... 371
Charcoal Pencils to Replace the Actual Cautery .... 372
Decoction of Sage in Profuse Sweating .... 372

